# Supplementary figures and images for: Deciphering the genetic landscape of tomato leaf curl New Delhi virus: Dynamic and region-specific diversity revealed by comprehensive sequence analyses
Source: PLoS One. 2025 Jul 17;20(7):e0326349. doi: 10.1371/journal.pone.0326349 (PMC12270167; doi:10.1371/journal.pone.0326349)

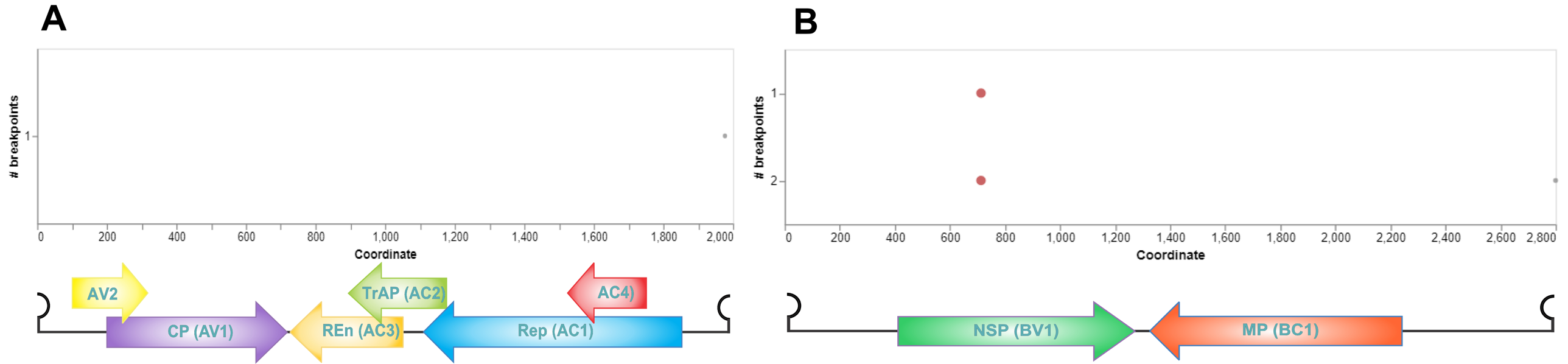

Supplement: S1 Fig — Well-supported breakpoints, identified by high AICc scores, are highlighted as bold red dots. Conversely, breakpoints with lower AICc scores are represented by smaller grey dots. The linear organization of the genomes for TA and TB is also depicted. (JPG) [file pone.0326349.s001.jpg]
