# Supplementary material for: Deciphering the genetic landscape of tomato leaf curl New Delhi virus: Dynamic and region-specific diversity revealed by comprehensive sequence analyses
Source: PLoS One. 2025 Jul 17;20(7):e0326349. doi: 10.1371/journal.pone.0326349 (PMC12270167; doi:10.1371/journal.pone.0326349)
Supplement: S2 Table — (DOCX) [file pone.0326349.s004.docx]

**S2 Table. Multiple recombination events detected by RDP in TA and TB genome components.**

| **Virus component** | **Accession number** | **Recombination event** | **Breakpoints** | | **Parents** | | **Detection Methods ^a^** | ***p*-value ^b^** |  |
| --- | --- | --- | --- | --- | --- | --- | --- | --- | --- |
| **TA** |  |  | **Begin** | **End** | **Major** | **Minor** |  |  |  |
|  | DQ116883 | 2 | 1006 | 1459 | KP195262 | MH577018 | BCGM**R**S3 | 3.47×10^−23^ |  |
|  |  |  | 1152 | 1415 | AJ620187 | KP195262 | BCGM**R**S3 | 3.86×10^−12^ |  |
|  | DQ989325 | 2 | 981 | 1381 | KC513822 | NC038471 | BCGM**R**S3 | 8.18×10^−39^ |  |
|  |  |  | 1753 | 2317 | KX467567 | KP195365 | GMRS**3** | 7.41×10^−14^ |  |
|  | JN587811 | 3 | 1218 | 1413 | KF551577 | MK069591 | BCGM**R**3 | 2.32×10^−14^ |  |
|  |  |  | 2120 | 2536 | MT085663 | AF102276 | CGM**R**3 | 8.74×10^−07^ |  |
|  |  |  | 2087 | 589 | Unknown | MW620973 | BCM**S**3 | 8.51×10^−05^ |  |
|  | JX460805 | 3 | 607 | 724 | MK336424 | KC960492 | BCGM**R**S3 | 8.12×10^−20^ |  |
|  |  |  | 607 | 1347 | DQ169056 | MK069591 | BCGMRS**3** | 9.73×10^−73^ |  |
|  |  |  | 748 | 1329 | KC460492 | KP191047 | GMR**S**3 | 3.71×10^−11^ |  |
|  | KF551576 | 2 | 251 | 1449 | DQ116880 | MH577015 | CGMR**3** | 7.57×10^−09^ |  |
|  |  |  | 1487 | 2356 | MK336424 | KP195262 | BCGMS**3** | 3.06×10^−26^ |  |
|  | KF551592 | 3 | 687 | 1422 | Unknown | KF551577 | BCGM**R**S3 | 9.59×10^−20^ |  |
|  |  |  | 1851 | 2089 | Unknown | KP178729 | BCGM**R**3 | 6.29×10^−15^ |  |
|  |  |  | 2470 | 2632 | MK069591 | KC513822 | BMRS**3** | 7.47×10^−15^ |  |
|  | KP195265 | 3 | 26 | 1427 | KP195265 | Unknown | BCGMRS**3** | 3.08×10^−42^ |  |
|  |  |  | 331 | 827 | Unknown | JN587811 | BCMRS**3** | 3.06×10^−10^ |  |
|  |  |  | 1113 | 1427 | Unknown | KP191047 | CGMS**3** | 1.25×10^−08^ |  |
|  | KX827602 | 2 | 948 | 1450 | KC513822 | Unknown | BCGM**R**S3 | 8.18×10^−39^ |  |
|  |  |  | 1884 | 2101 | LN908935 | Unknown | BCGM**R**S3 | 3.28×10^−16^ |  |
|  | KY420139 | 3 | 2739 | 141 | Unknown | DQ989325 | **C**GMR3 | 5.40×10^−08^ |  |
|  |  |  | 1764 | 2331 | KF551577 | KY780202 | CMRS**3** | 3.37×10^−06^ |  |
|  |  |  | 564 | 2331 | Unknown | AM849548 | BCGMRS**3** | 8.05×10^−68^ |  |
|  | MH328254 | 2 | 135 | 1350 | Unknown | MH328255 | CMR**S**3 | 3.16×10^−05^ |  |
|  |  |  | 1360 | 2372 | MN630287 | Unknown | BCGMR**S**3 | 4.03×10^−14^ |  |
|  | MH465599 | 3 | 2064 | 34 | AM747291 | Unknown | BCGMS**3** | 6.32×10^−23^ |  |
|  |  |  | 2425 | 2648 | KY780207 | MH475911 | BCG**M**RS3 | 6.44×10^−24^ |  |
|  |  |  | 450 | 1035 | KY780201 | Unknown | GMRS**3** | 9.60×10^−06^ |  |
|  | MH577018 | 2 | 1046 | 1451 | Unknown | KP195262 | BCGM**R**S3 | 3.10×10^−20^ |  |
|  |  |  | 34 | 850 | Unknown | U15015 | BCGMRS**3** | 5.37×10^−07^ |  |
|  | MK069591 | 3 | 614 | 741 | MK336424 | KC960492 | BCGM**R**S3 | 8.17×10^−20^ |  |
|  |  |  | 1245 | 1420 | Unknown | KC960492 | GMRS**3** | 3.71×10^−11^ |  |
|  |  |  | 2440 | 2642 | KY780202 | MH475911 | CG**R**S3 | 7.34×10^−09^ |  |
|  | MK336424 | 4 | 34 | 708 | MW426871 | Unknown | BCGM**R**S3 | 3.89×10^−21^ |  |
|  |  |  | 344 | 708 | KP191047 | KP195265 | BCGMR**3** | 8.07×10^−16^ |  |
|  |  |  | 1050 | 1963 | MW426871 | Unknown | BGMS**3** | 4.81×10^−11^ |  |
|  |  |  | 2471 | 2733 | MK551587 | KP191047 | BCGMS**3** | 2.29×10^−22^ |  |
|  | MN630279 | 2 | 2055 | 2168 | MK883715 | MN630277 | CGMR**3** | 1.43×10^−07^ |  |
|  |  |  | 2328 | 2709 | MN630294 | MH328257 | CGMS**3** | 1.59×10^−22^ |  |
|  | MN630280 | 2 | 2052 | 2165 | MK883715 | MN630277 | BCGMRS**3** | 1.43×10^−07^ |  |
|  |  |  | 2336 | 2728 | MN630294 | MH328257 | BCGMRS**3** | 1.59×10^−22^ |  |
|  | MN630281 | 2 | 2055 | 2168 | MK883715 | MN630277 | BCGMRS**3** | 1.43×10^−07^ |  |
|  |  |  | 2339 | 2731 | MN630294 | MH328257 | BCGMRS**3** | 1.59×10^−22^ |  |
|  | MN630282 | 2 | 2055 | 2168 | MK883715 | MN630277 | BCGMRS**3** | 1.43×10^−07^ |  |
|  |  |  | 2339 | 2731 | MN630294 | MH328257 | BCGMRS**3** | 1.59×10^−22^ |  |
|  | MN630287 | 2 | 383 | 2284 | Unknown | MN630294 | BCGMR**S**3 | 7.77×10^−16^ |  |
|  |  |  | 2051 | 2166 | MK883715 | MN630277 | CGMR**3** | 1.43×10^−07^ |  |
|  | MN630290 | 2 | 1968 | 451 | MN630291 | Unknown | BCM**S**3 | 4.12×10^−17^ |  |
|  |  |  | 191 | 462 | MN630290 | Unknown | CMRS**3** | 9.32×10^−06^ |  |
|  | NC038471 | 2 | 685 | 1422 | Unknown | KF551577 | BCGM**R**S3 | 9.59×10^−20^ |  |
|  |  |  | 1801 | 2096 | Unknown | KP178729 | BCGM**R**3 | 6.29×10^−15^ |  |
|  | | | | | | | | | |
| **TB** | DQ020490 | 2 | 1273 | 2139 | MH465600 | MG597207 | BCGMRS**3** | 1.24×10^−18^ |  |
|  |  |  | 2296 | 2566 | Unknown | KC545813 | BCGM**S**3 | 5.78×10^−16^ |  |
|  | GU112089 | 2 | 283 | 2066 | GU112083 | HM803117 | BCGRMS**3** | 1.66×10^−38^ |  |
|  |  |  | 1433 | 2066 | HM803117 | AY158080 | BC**G**RMS3 | 3.79×10^−12^ |  |
|  | HQ141674 | 2 | 162 | 245 | HM803117 | KF577601 | BG**R**M3 | 4.34×10^−09^ |  |
|  |  |  | 2340 | 33 | Unknown | KF577602 | BCGRM**S**3 | 1.24×10^−20^ |  |
|  | JN208137 | 2 | 1164 | 2156 | KY780206 | AM286435 | CGMS**3** | 5.24×10^−15^ |  |
|  |  |  | 2273 | 2461 | KT948073 | AM778833 | BCGM**R**S3 | 6.39×10^−06^ |  |
|  | KF515623 | 3 | 94 | 826 | KF577603 | Unknown | BCGM**R**S3 | 2.54×10^−19^ |  |
|  |  |  | 120 | 412 | KF577603 | Unknown | C**G**MR**S**3 | 1.99×10^−36^ |  |
|  |  |  | 1593 | 2286 | Unknown | HM159455 | BCGMR**S**3 | 1.24×10^−20^ |  |
|  | KF577603 | 3 | 110 | 405 | Unknown | KY780206 | BCGM**R**S3 | 5.56×10^−24^ |  |
|  |  |  | 444 | 918 | MF967023 | Unknown | CMR**S**3 | 2.05×10^−11^ |  |
|  |  |  | 949 | 2341 | Unknown | HM159455 | BCGMR**S**3 | 1.24×10^−20^ |  |
|  | KF577604 | 2 | 1 | 355 | KY780208 | LN845963 | BCMRS**3** | 2.06×10^−21^ |  |
|  |  |  | 987 | 2129 | KY780208 | Unknown | BC**M**RS3 | 3.99×10^−15^ |  |
|  | KF577605 | 2 | 265 | 388 | KJ778695 | LN845937 | BCGMRS**3** | 5.30×10^−10^ |  |
|  |  |  | 878 | 2161 | AY438563 | LN845936 | BCMRMS**3** | 1.28×10^−33^ |  |
|  | KY420144 | 2 | 40 | 517 | MW426952 | MT592862 | BCGMR**S**3 | 5.21×10^−09^ |  |
|  |  |  | 2274 | 2489 | KT948073 | AM778833 | BCGM**R**S3 | 6.37×10^−08^ |  |
|  | KY780203 | 2 | 1032 | 2151 | KY780206 | KY780208 | BCGMRS**3** | 3.55×10^−40^ |  |
|  |  |  | 2273 | 2461 | KT948073 | AM778833 | BCGM**R**S3 | 6.37×10^−06^ |  |
|  | KY780214 | 2 | 600 | 1383 | KY780212 | Unknown | BCGMRS**3** | 3.90×10^−19^ |  |
|  |  |  | 656 | 1011 | Unknown | HG316126 | GMRS**3** | 9.74×10^−08^ |  |
|  | KY933712 | 2 | 1524 | 1964 | KY933710 | KY933711 | BC**G**MS3 | 2.77×10^−32^ |  |
|  |  |  | 2559 | 2640 | MH520665 | Unknown | BCG**R**3 | 3.06×10^−11^ |  |
|  | MG597209 | 3 | 286 | 1868 | HG316126 | MG597207 | BCGRMS**3** | 4.07×10^−11^ |  |
|  |  |  | 2267 | 2478 | MW538660 | GU112083 | BCM**S**3 | 6.23×10^−06^ |  |
|  |  |  | 2518 | 2610 | MG597211 | Unknown | BGR**S**3 | 9.50×10^−27^ |  |
|  | MH465600 | 2 | 1399 | 2124 | Unknown | KY780214 | CMRS**3** | 1.12×10^−08^ |  |
|  |  |  | 2321 | 2566 | Unknown | KC545813 | BCGM**S**3 | 5.78×10^−16^ |  |
|  | MT592859 | 3 | 484 | 835 | LT168870 | HG316126 | CGMRS**3** | 1.39×10^−10^ |  |
|  |  |  | 1404 | 2176 | Unknown | LN845943 | BCGMRS**3** | 1.98×10^−25^ |  |
|  |  |  | 2237 | 2565 | LN845949 | Unknown | BCGMR**S**3 | 7.77×10^−10^ |  |
|  | MT592862 | 3 | 484 | 835 | LT168870 | HG316126 | CGMRS**3** | 1.39×10^−10^ |  |
|  |  |  | 1404 | 2176 | Unknown | LN845943 | BCGMRS**3** | 1.98×10^−25^ |  |
|  |  |  | 2237 | 2621 | LN845949 | Unknown | BCGMRS**3** | 1.53×10^−10^ |  |
|  | MT592863 | 3 | 484 | 835 | LT168870 | HG316126 | CGMRS**3** | 1.39×10^−10^ |  |
|  |  |  | 1404 | 2176 | Unknown | LN845943 | BCGMRS**3** | 1.98×10^−25^ |  |
|  |  |  | 2237 | 2621 | LN845949 | Unknown | BCGMRS**3** | 1.53×10^−10^ |  |
|  | LN845940 | 3 | 510 | 1009 | Unknown | LN845961 | BCGM**S**3 | 1.47×10^−21^ |  |
|  |  |  | 510 | 1990 | MT592862 | MW426952 | BCGMRS**3** | 6.51×10^−35^ |  |
|  |  |  | 2237 | 2565 | LN845949 | Unknown | BCGMR**S**3 | 7.77×10^−10^ |  |
|  | LN845944 | 2 | 2498 | 425 | MW426952 | MT592862 | BCGMR**S**3 | 5.21×10^−09^ |  |
|  |  |  | 1403 | 2313 | LN845961 | MT592863 | BCGMS**3** | 5.53×10^−30^ |  |
|  | LT168854 | 2 | 264 | 489 | KJ778695 | LN845937 | BCGMRS**3** | 6.77×10^−10^ |  |
|  |  |  | 1559 | 1809 | LT168861 | LT168856 | BCG**R**S3 | 1.44×10^−19^ |  |
|  | LT168869 | 2 | 1528 | 2047 | LN845961 | LT168877 | BC**G**MRS3 | 2.87×10^−24^ |  |
|  |  |  | 2273 | 2458 | KT948073 | AM778833 | BCGM**R**S3 | 6.39×10^−08^ |  |
|  | LT168870 | 2 | 1528 | 2047 | LN845961 | LT168877 | BC**G**MRS3 | 2.87×10^−24^ |  |
|  |  |  | 2273 | 2458 | KT948073 | AM778833 | BCGM**R**S3 | 6.39×10^−08^ |  |
|  | LT168873 | 3 | 629 | 2082 | LT168870 | LT168877 | BCGMRS**3** | 2.89×10^−63^ |  |
|  |  |  | 675 | 1289 | HG316126 | KX951456 | **C**MRS3 | 9.71×10^−04^ |  |
|  |  |  | 2272 | 2458 | KT948073 | AM778833 | BCGMRS**3** | 5.68×10^−06^ |  |
|  | MK336425 | 2 | 245 | 819 | KF577605 | EF620535 | CGMRS**3** | 1.20×10^−10^ |  |
|  |  |  | 2329 | 2554 | EF408038 | LT168879 | BC**G**MRS3 | 8.52×10^−35^ |  |

^a^ B, Bootscan; C, Chimaera; G, GeneConv; L, LARD; M, MaxChi; P, Phylpro; R, RDP; S, SisScan; 3, 3SEQ.

^b^ The lowest *p*-value corresponds to the recombination program (bold) is mentioned.

Abbreviation used in the Table are tomato leaf curl New Delhi virus DNA-A (TA) and DNA-B (TB).
