# Supplementary material for: Deciphering the genetic landscape of tomato leaf curl New Delhi virus: Dynamic and region-specific diversity revealed by comprehensive sequence analyses
Source: PLoS One. 2025 Jul 17;20(7):e0326349. doi: 10.1371/journal.pone.0326349 (PMC12270167; doi:10.1371/journal.pone.0326349)
Supplement: Attachment — Submitted filename: comments.doc [file pone.0326349.s006.doc]

**Comments on manuscript**

Title:  Deciphering the Genetic Landscape of ToLCNDV: Dynamic and Region-Specific Diversity Revealed by Comprehensive Analyses

ToLCNDV is one of the important viral pathogens worldwide. Selection of problem is good. Manuscript is written nicely.

I have gone through the manuscript, the following corrections were raised and few highlighted in manuscript.

Quality of images of Fig 3 and Fig 4 should be improved.

Include all crops also and their distribution across the continents.

Include association of satellite molecules.

The outcome the manuscript useful to virologists and breeders

I recommend this manuscript for publication with major corrections.
