# Supplementary material for: Deciphering the genetic landscape of tomato leaf curl New Delhi virus: Dynamic and region-specific diversity revealed by comprehensive sequence analyses
Source: PLoS One. 2025 Jul 17;20(7):e0326349. doi: 10.1371/journal.pone.0326349 (PMC12270167; doi:10.1371/journal.pone.0326349)
Supplement: Attachment — Submitted filename: Response to Editor and Reviewers_R1.docx [file pone.0326349.s008.docx]

***Response to Reviewers***

Authors sincerely thank the reviewer for their insightful and constructive feedback. Authors tried to address each comment in detail (responses in red font, below), and corresponding revisions are marked with track changes in the manuscript. All line numbers referenced pertain to the track-changed version.

**Journal Requirements:**

1. Please note that your Data Availability Statement is currently missing the DOI/accession number of each dataset OR a direct link to access each database. If your manuscript is accepted for publication, you will be asked to provide these details on a very short timeline. We therefore suggest that you provide this information now, though we will not hold up the peer review process if you are unable.

Response: Authors added webpage address (https://blast.ncbi.nlm.nih.gov/) from where accession numbers can be accessed.

2. Please remove your figures/ from within your manuscript file, leaving only the individual TIFF/EPS image files. These will be automatically included in the reviewer’s PDF

Response: Authors removed all the figures from the manuscript file.

3. Thank you for the information about your Google map images. While Google does allow the reuse of these images, there are a few restrictions which are not compatible with the Creative Commons Attribution license.

Response: Authors replaced the Google Maps image with a map sourced from NASA’s public database (https://eol.jsc.nasa.gov/), which is approved for use by PLOS and free of copyright restrictions

**Reviewer Comments:**

***REVIEWER 1***

Query 1.This MS was made based on totally online data available in GenBank, no original work.

Response: We appreciate the reviewer’s observation. We respectfully disagree with the notion that the manuscript lacks originality. The novelty of our study does not stem from the generation of new sequence data, but rather from the original analysis, interpretation, and biological insight we provide using existing datasets. Our work addresses previously unexplored questions regarding genetic diversity, evolutionary dynamics, and recombination landscape of ToLCNDV, a virus of growing global concern. Our work offers original contributions through the application of genetic diversity indices, leading to new interpretations of previously unexplored biological questions.

Q2. Why this work was not done by using ToLCV sequences submitted from KSA?
All the data is used from GenBank and nothing new data in this MS, all are old.

Q3. In the introduction part, authors have mentioned that many countries but no KSA? Why?

Q4. In materials and methods section: From these datasets, sub-datasets were generated for each open reading frame (ORF). Additionally, sequences were categorized by country of origin, with further division into their respective ORFs to facilitate country-specific analyses. Where is KSA?

Response to Q2, Q3, and Q4: We appreciate the reviewer’s query regarding the use of ToLCNDV sequences from KSA. The study focuses on a comprehensive analysis of ToLCNDV, which, to our knowledge, has not been reported in the KSA, and thus, no ToLCNDV sequences from KSA are available in GenBank or elsewhere. As we mentioned in our response to query 1 that the originality of this work lies in its novel bioinformatics approach and the analysis of these data to generate new insights related to GDIs. We are open to further emphasizing the novelty of our approach or addressing specific suggestions to strengthen the manuscript.

Q5. Results section: The figures and Tables: No data from KSA at all
Some figures are not with high resolution and should be replaced with high resolution fig.
The phylogenetic tree images are not at all visible.

Response to Q5: The authors have enhanced the resolution of the figures in the revised manuscript. However, due to the complexity of the phylogenetic figure's data, further refinements were not possible. As an alternative, a color scheme was implemented to improve clarity and ease of identification.

Q6. Authors should provide the genetic diversity/similarity % in a table.

Response to Q6: Thank you for your valuable suggestion. The authors did perform a % similarity analysis using the SDT v1.2 and MEGA11 tools, which generate pairwise similarity matrices based on nucleotide alignments. However, due to the large number of sequences included in our dataset, the resulting similarity matrix was extensive, making it impractical to present either as a figure or a table within the manuscript. Moreover, the primary focus of our study was on the broader evolutionary dynamics, phylogenetic relationships, and recombination patterns, rather than detailed pairwise identity comparisons. Therefore, while the SDT analysis supported our findings, its inclusion was deemed unnecessary for the scope of this manuscript. We appreciate the reviewer’s understanding in this matter.

Q7. Figure 3. The phylogenetic dendrogram of selected sequences of ToLCNDV, TA (A) and TB (B), based on their respective full-length sequences. The phylogenetic tree was constructed with Maximum-Likelihood (ML) algorithm in MEGA11 with 1000 bootstrap values. Geographic origin (country) of the isolates is indicated by the coloured circle. Why not some sequences from KSA was used in this study?

Response to Q7: ToLCNDV has not been reported from KSA (please refer to our response against Q2-Q4).

Q8. RDP generates some important figures and data and this MS lacking those images.
Recombination breakpoints (start and end points) in ToLCNDV genome should be reflected in the image.

Response to Q8: We appreciate the reviewer's comment regarding the presentation of the RDP analysis results. Due to the exceptionally high frequency and complex nature of detected recombination events, including numerous overlapping breakpoints across hundreds of sequences (summarized in Table 2 and S2 Table), a graphical representation of each event was not feasible. Traditional visualization methods, such as genome maps detailing each breakpoint, would result in an uninterpretable figure that would not effectively convey the overall recombination patterns. Therefore, the comprehensive RDP outputs are provided in tabular format to allow for detailed examination of the identified recombination events.

Q9. All the figures and Data is made and generated by softwares, there is no novel findings in this MS and does not reflect the originality of the work.

Response to Q9: We respectfully disagree with the notion that the manuscript lacks originality. The novelty of our study does not stem from the generation of new sequence data, but rather from the original analysis, interpretation, and biological insight we provide using existing datasets. Our work addresses previously unexplored questions regarding the genetic diversity, evolutionary dynamics, and recombination landscape of Tomato leaf curl New Delhi virus (ToLCNDV), a virus of growing global concern.

- While it is true that bioinformatics tools were used to generate figures and perform analyses—as is standard practice in genomic studies—the strength and innovation of our manuscript lie in:
- The integrated approach applied across multiple genomic regions and populations;
- The scale and comprehensiveness of the recombination and phylogenetic analyses across a diverse set of ToLCNDV isolates;
- The interpretation of recombination events in relation to functional genomic regions, providing new insights into how ToLCNDV may adapt and evolve;
- The identification of evolutionary patterns not previously reported in the context of this virus.

These aspects represent novel contributions to the field, offering meaningful perspectives on virus evolution, potential diagnostic targets, and disease management strategies. Thus, while the data are publicly available, our scientific interpretation, synthesis, and conclusions are original and advance the understanding of ToLCNDV in a way that has not been previously documented.

Q10. The accession numbers, name of the viruses’ acronyms are not available in this MS.

Response to Q10: Please see the S1 Table for accession numbers of the used ToLCNDV sequences.

Q11. Discussion: This study used stratified random sampling to ensure each country was represented Proportionally to its population size, thus mitigating over- or under-representation.

Where is KSA data? If the funding agency from KSA then KSA data should be used so that the generated information will be valuable for KSA.

Response to Q11: Since ToLCNDV is not yet reported from KSA, so, the absence of KSA-specific data is due to the current lack of documented infections rather than an oversight in sampling or analysis.

The generated data from this work is not as much as useful for scientific community as well as for KSA.
There are some grammatical errors were observed in the text.
Conclusion: Notably, Asian isolates displayed significantly, higher recombination rates, indicating location-dependent evolution. Again, KSA data missing.

Response to Q: Typo and grammatical errors have been rectified from the revised script.

**Reviewer #2**

I have critically reviewed the manuscript title “Deciphering the Genetic Landscape of ToLCNDV: Dynamic and Region-Specific Diversity Revealed by Comprehensive Analyses”, which describes the genetic diversity and evolutionary tracks of the most important tomato infecting bipartite begomovirus ToLCNDV. The manuscript is about very important issue of devastating begomoviruses which belong the largest genus of plant infecting viruses with wide host range. The manuscript utilized the advanced bioinformatics softwares to analyze the nucleotide mutation rate and recombination events in more than 650 virus complete genomes. The manuscript is well written describing the importance of study supported with update review of literature. Methodology of experiments are well explained to reproduce the experimental results. The results and discussions are comprehensively described. Although there are few typo errors which can corrected by careful reading of the manuscript.
I would recommend the acceptance of manuscript for publication in its present form.

Response: The authors sincerely thank the reviewer for dedicating his/her valuable time to evaluating the manuscript and for his/her constructive and encouraging feedback.

**Reviewer #3**

**:** This paper studied “Deciphering the Genetic Landscape of ToLCNDV: Dynamic and Region-Specific Diversity Revealed by Comprehensive Analyses”. In general, this is a well written paper and the results support the main conclusion. Here, I would like to give few suggestions to further improve the manuscript:
1, The abstract can be more precise.

Response: We thank the reviewer for their careful reading of our manuscript and their suggestion to improve the abstract. While we aimed to keep the abstract concise to adhere to journal guidelines, we also strove to ensure it remained precise and informative. Nevertheless, we tried our best to concise the abstract as per suggestion. Please see the revised version of the script.

2, Briefly discuss how recombination may impact virulence, host adaptation, or resistance breakdown.

Response to Q2: The mentioned points have been added in the revised script. Please see Page 16 and Lane 476-483 of the revised script.

3, Briefly discuss why AC4 and Rep show diversifying selection while other genes are under purifying selection.

Response to Q3: The query asked has been addressed. Please see Page 23, Line 545-550 of the revised script.

4, Emphasize the importance of integrated vector management (IVM) to limit viral spread.

Response to Q4: IVM along with its importance has been added to the script. Please see Page 17, Lane 509-514 in the revised script.

**Reviewer #4**

ToLCNDV is one of the important viral pathogens worldwide. Selection of problem is good. Manuscript is written nicely.
I have gone through the manuscript, the following corrections were raised and few highlighted in manuscript.

Q1. Quality of images of Fig 3 and Fig 4 should be improved.

Response to Q1: Both the figures have been improved in the revised script. However, due to the complexity and large data of the phylogenetic figure, further refinements were not possible.

Q2. Include all crops also and their distribution across the continents.

Response to Q2: The query has been addressed. Please see Page 6, line 209-217 of the revised script.

Q3. Include association of satellite molecules.

Response to Q3: This point has already been mentioned in the script (page 2, line 78-79).
